# Editorial: insights in cancer endocrinology 2021

**DOI:** 10.3389/fendo.2022.987764

**Published:** 2022-07-29

**Authors:** Claire M. Perks, Antonino Belfiore

**Affiliations:** ^1^ Bristol Medical School, Department of Translational Health Sciences, University of Bristol, Bristol, United Kingdom; ^2^ Endocrinology Unit, Department of Clinical and Experimental Medicine, University of Catania, Garibaldi-Nesima Hospital, Catania, Italy

**Keywords:** cancer, cell signalling, prostate, breast, neuroendocrine tumors

This Research Topic entitled “Insights in Cancer Endocrinology: 2021” provides a collection of work highlighting important issues in the field, for example, furthering our understanding of important cell signalling pathways and optimization of treatment options in different cancers. Panigoro et al. report on a multicentre, case-control study conducted in six public referral hospitals in Indonesia and assessed the use of triglyceride glucose index (TyG) as a reliable and cost-effective surrogate marker of insulin resistance. The authors found it to be associated with the risk of breast cancer in a non-linear fashion. Mutations in the breast and ovarian cancer susceptibility gene (BRCA1) are also associated with an increased risk of developing cancers, including those of the breast and ovary. Werner’s review emphasizes the emerging non-genomic roles of BRCA1, for example being an important regulator of endocrine and metabolic axes, including the insulin/IGF1 signalling pathway. BRCA1 mutations are also correlated with metabolic disorders, such as metabolic syndrome and diabetes. A mini-review written by Bulatowitcz et al. discusses the role of another important regulator of cell behavior in cancer progression, the insulin-like growth factor 1 receptor (IGF-1R). The IGF-1R has mostly been described as pro-tumorigenic, but the authors highlight that both pro- and anti-tumorigenic roles have been described mainly in mouse models of breast cancer and they provide hypotheses to support these conflicting data [IN PRESS].

Prostate cancer (PCa) remains the second most common cancer in men globally and Wang et al. describe the ‘burden’ of PCa in China from 1990- 2019. Whilst the incidence in China is still relatively low compared with other countries, it appears to be on the increase and the rates and disease burden amongst the provinces in China are very different. The authors conclude that based on this, implementing more targeted and effective strategies should be considered [IN PRESS]. In terms of new therapeutic options for men with advanced PCa, Licitra et al. have written a review highlighting the limited approaches available. They report that a better understanding in recent years of for example, the role of the tumor micro-environment, has led to the emergence of novel therapeutic approaches.

There are many adverse side effects of cancer therapies and a vitally important one is highlighted by Eugeni et al. who reviewed current available approaches to protect or restore fertility in young prepubertal male patients, before they undergo potentially damaging treatments, such as radiotherapy[IN PRESS]. In addition, immune checkpoint inhibitors are currently now used to treat cancer patients and Barnabei et al. have reported that a rare side-effect of these drugs is central diabetes insipidus (CDI) and to provide better management of the adverse effects and the specific drugs that trigger it, they describe a new, improved grading system for CDI.

Von Hippel-Lindau (VHL) disease is a hereditary condition associated with tumors occurring in multiple organs, including the brain, spine, eyes, kidneys, pancreas, adrenal glands, inner ears, reproductive tract, liver, and lung, and is caused by mutations in a tumor suppressor gene called *VHL*. Mathó et al. characterise two novel variants of this gene, to help understand how they may affect the function and fate of VHL protein and impact on disease progression.

Neuroendocrine tumors (NETs) are rare cancers and can develop in different locations, as neuroendocrine cells are found in most organs of the body. Puliani et al. have written a mini review to provide a new vision for the potential increased use, safety and prognostic and predictive factors of two radiopharmaceuticals, ^177^Lu-DOTATATE and ^90^Y-DOTATOC in peptide receptor radionuclide therapy (PPRT) for NETs. Contarino et al. focused on medullary thyroid carcinoma and in their case series and review of the literature they asked the question: “Is encapsulated medullary carcinoma associated with a better prognosis?”. Cui et al. retrospectively analysed clinical data from patients with local-regional recurrence of pheochromocytoma/paraganglioma and reported on their characteristics, risk factors and outcome. Giuffrida et al. analyzed the methylation profile from eleven growth hormone-secreting (GH-omas) and ten non-functioning (NF-omas) pituitary adenomas and report that the epigenetic landscape of these tumors is complicated. Whilst hypermethylation is considerably more prevalent in NFPA compared with GH-omas, its contribution to the disease is still to be determined. Furthermore, Mangili et al. performed *in vitro* studies to examine if cabergoline in combination with mTOR inhibitors may be a potential treatment specifically for patients with β-arrestin2 expressing pituitary neuroendocrine tumors or other subtypes also resistant to conventional therapy [IN PRESS].

It has been our pleasure to host this exciting Research Topic and we thank all the authors for their excellent contributions.

## Author contributions

CP wrote and AB & CP read, commented and edited the article. All authors contributed to the article and approved the submitted version.

## Conflict of interest

The authors declare that the research was conducted in the absence of any commercial or financial relationships that could be construed as a potential conflict of interest.

## Publisher’s note

All claims expressed in this article are solely those of the authors and do not necessarily represent those of their affiliated organizations, or those of the publisher, the editors and the reviewers. Any product that may be evaluated in this article, or claim that may be made by its manufacturer, is not guaranteed or endorsed by the publisher.

